# Inner-shell electrons enable both high power and energy densities

**DOI:** 10.1093/nsr/nwaf139

**Published:** 2025-04-10

**Authors:** Yi Jiang, Kaiwen Zeng, Zhe Yang, Tianrui Li, Yunting Zhang, Bingjie Wang, Huisheng Peng

**Affiliations:** State Key Laboratory of Molecular Engineering of Polymers, Department of Macromolecular Science, Institute of Fiber Materials and Devices, and Laboratory of Advanced Materials, Fudan University, Shanghai 200438, China; State Key Laboratory of Molecular Engineering of Polymers, Department of Macromolecular Science, Institute of Fiber Materials and Devices, and Laboratory of Advanced Materials, Fudan University, Shanghai 200438, China; State Key Laboratory of Molecular Engineering of Polymers, Department of Macromolecular Science, Institute of Fiber Materials and Devices, and Laboratory of Advanced Materials, Fudan University, Shanghai 200438, China; State Key Laboratory of Molecular Engineering of Polymers, Department of Macromolecular Science, Institute of Fiber Materials and Devices, and Laboratory of Advanced Materials, Fudan University, Shanghai 200438, China; State Key Laboratory of Molecular Engineering of Polymers, Department of Macromolecular Science, Institute of Fiber Materials and Devices, and Laboratory of Advanced Materials, Fudan University, Shanghai 200438, China; State Key Laboratory of Molecular Engineering of Polymers, Department of Macromolecular Science, Institute of Fiber Materials and Devices, and Laboratory of Advanced Materials, Fudan University, Shanghai 200438, China; State Key Laboratory of Molecular Engineering of Polymers, Department of Macromolecular Science, Institute of Fiber Materials and Devices, and Laboratory of Advanced Materials, Fudan University, Shanghai 200438, China

**Keywords:** energy conversion, radiative recombination, power density, energy density

## Abstract

Energy supply technologies are mainly based on lithium-ion batteries, which are approaching their theoretical power density of 1901 W⋅L^−1^ and energy density of 568 Wh⋅kg^−1^ with the use of outer-shell electrons. The above limitations are recognized as a critical problem in energy science and have severely hindered the development of modern electronics. Herein, we explore the potential of utilizing the transition of inner-shell electrons for energy conversion. Flexible Atomic Code on the radiative recombination of highly charged xenon ions with free electrons shows a radiative power output of 2.35 × 10^5^ W⋅L^−1^ and an energy release of 8.65 × 10^7^ Wh⋅kg^−1^ under specific ion and electron density conditions. This finding suggests a promising strategy to discover new energy technologies with extremely high power and energy densities in the future.

## Teaser

A novel energy conversion approach using inner-shell electron transitions in xenon ions achieves both high power and energy densities.

## INTRODUCTION

The growing global energy demand, particularly for portable electronics and electric vehicles, has intensified the need for energy solutions with rapid power outputs and high energy densities. In response, extensive research over the past few decades has been focused on advancing electrode materials [1], developing novel electrolyte systems [2] and optimizing cell architectures [3] to enhance performance. For instance, power densities of lithium-ion batteries have been significantly enhanced from 200 W⋅L^−1^ upon commercialization in 1991 [4] to 750 W⋅L^−1^ currently [5], and the theoretical power density is reported as ∼1901 W⋅L^−1^ [6]. More importantly, their energy densities have been increased from 80 to 360 Wh⋅kg^−1^, approaching the theoretical energy density of ∼568 Wh⋅kg^−1^. However, the power and energy densities of lithium-ion batteries will obviously lag behind the boom of modern electronics and information technology even after they reach the theoretical values. While challenging, it is critical to discover new energy technologies for much higher power and energy densities.

**Figure 1.**
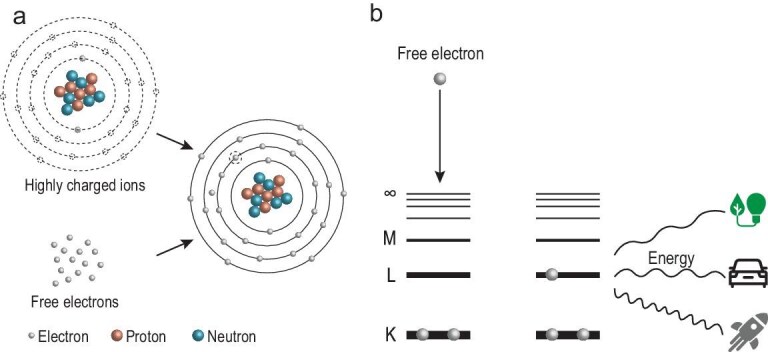
(a) Schematic diagram of inner-shell electron transitions. (b) Radiative recombination process in He-like ions.

For batteries, energy is primarily derived from outer-shell electron reactions. In typical chemical bonding processes, energy change is primarily determined by the interactions in outer-shell electrons (Fig. S1) [7]. For instance, the formation of a lithium-fluorine bond releases 5.9 eV, making it one of the strongest bonds. In contrast, inner-shell electrons are much more tightly bound to the nucleus by stronger Coulomb forces, with binding energies reaching tens of kiloelectronvolts (keV) [8]. For instance, a K-shell electron in a xenon (Xe) atom has a binding energy of ∼34.6 keV [9], which is thousands of times greater than the energy involved in outer-shell chemical reactions. Additionally, the abundance of inner-shell electrons in heavy elements further boosts the potential energy output, making inner-shell electron transition a promising avenue for high-power and high-energy-density applications (Fig. 1a). However, to the best of our knowledge, inner-shell electron transitions have not yet been explored for energy conversion.

Herein, we report the potential of inner-shell electron transitions for energy conversion. Xe, a noble gas with an atomic number of 54 is selected for the demonstration due to its abundant inner-shell electrons and ready availability for practical application. With the use of Flexible Atomic Code (FAC), we simulate the radiative recombination of highly charged Xe^52^⁺ with free electrons. The calculation results show that energy release falls in the range of 2–10 keV, with a dominant emission peak between 8 and 10 keV, as Xe ion charge states evolve from Xe^52^⁺ to Xe^44^⁺. Under moderate electron and ion densities, the system achieves an extremely high radiation power density of 2.35 × 10^5^ W⋅L^−1^ and an energy density of 8.65 × 10^7^ Wh⋅kg^−1^.

## RESULTS AND DISCUSSION

### Radiative recombination cross sections of highly charged Xe ions

To investigate the energy release by inner-shell electrons, we first examine the electron capture by highly charged ions. Under our working conditions with moderate electron density, radiative recombination dominates over non-radiative processes such as Auger and three-body recombination, ensuring efficient energy conversion through photon emission. Radiative recombination is a non-resonant and one-step recombination process, in which a free electron recombines with an ion, emitting excess energy in the form of a photon [10]:


(1) \begin{eqnarray*}
{X^{q + }} + {e^ - } \to {X^{\left( {q - 1} \right) + }} + hv,
\end{eqnarray*}

where *X^q^^+^* and *X^(q^⁻^1)+^* represent the ion species before and after electron capture, respectively. The photon energy *hν* is given by:


(2) \begin{eqnarray*}
h\nu = {E_e} + {E_b}\left( {nl} \right),
\end{eqnarray*}

where *E_e_* is the kinetic energy of the free electron and *E_b_(nl)* is the binding energy of the state in which the free electron is captured. The process is illustrated in Fig. 1b.

The FAC is a comprehensive package for calculating various atomic radiative and collisional processes, including collisional excitation, photoionization, autoionization, and radiative and dielectronic recombination [11–13]. In the FAC, the radiative recombination cross section is derived from the photoionization cross section using the Milne relation. Photoionization cross sections are computed for electron energies *E_e_* < 10*E_th_*, where *E_th_* is the ionization threshold for the corresponding shells. For *E_e_* > 10*E_th_*, a simplified formula proposed by Verner *et al.* [14] is used:


(3) \begin{eqnarray*}
{\sigma _{PI}}( {{E_e}} ) = {\sigma _0}{\left( {\frac{{1 + b}}{{\sqrt x + b}}} \right)^p}{x^{ - 3.5 - l + \frac{p}{2}}},
\end{eqnarray*}

where *x = (E_0_ + E_e_)/E_0_, l* is the orbital angular momentum of the photoionized shell, and *σ_0_, E_0_, p* and *b* are fit parameters.

At high energies, both photoionization and radiative recombination cross sections of highly charged ions demonstrate closer conformity to hydrogenic approximations. Therefore, the procedure employed by FAC, which involves exact calculations at low energies and high-energy extrapolation based on hydrogenic approximation, is well suited for highly charged ions. Here, we use the FAC to calculate energy levels and radiative recombination cross sections from Xe^52+^ (He-like) to Xe^44+^ (Ne-like). The selection of Xe^44+^ as the final state is based on its neon-like configuration with a completed L shell, which represents a relatively stable electronic structure. The electron capture processes to L shell during this transition can effectively demonstrate the substantial energy release capability of inner-shell electrons. Electron energies from 1 to 1000 eV are considered to provide a comprehensive understanding of the recombination process across different energy regimes.

### Radiative recombination rate coefficients of highly charged Xe ions

The radiative recombination cross section is a key parameter in determining the total recombination rate, which represents the photon emission probability at specific energies. The radiative recombination rate coefficients for Xe^52+^ to Xe^45+^ as a function of electron energy are shown in Table 1, ranging from 1 to 1000 eV. The calculated rate coefficients exhibit the expected inverse relationship with electron energy, consistent with the physical principle that the probability of electron captures decreases at higher collision energies.

**Table 1.** Radiative recombination rate coefficients for Xe ions (Xe^52+^ to Xe^45+^) at different electron energies.

	Radiative recombination rate coefficients (10⁻^10^ cm^3^⋅s⁻^1^)							
	
Electron energy (eV)	Xe^52+^	Xe^51+^	Xe^50+^	Xe^49+^	Xe^48+^	Xe^47+^	Xe^46+^	Xe^45+^
1	109.481	107.987	103.874	193.298	448.085	413.933	380.816	139.167
100	10.525	10.371	9.952	18.482	42.750	39.382	36.115	13.150
200	7.110	6.997	6.700	12.410	28.619	26.273	24.003	8.700
300	5.571	5.476	5.235	9.674	22.252	20.368	18.547	6.697
400	4.648	4.564	4.356	8.033	18.435	16.831	15.283	5.499
500	4.017	3.941	3.755	6.913	15.832	14.422	13.061	4.685
600	3.533	3.482	3.313	6.089	13.920	12.653	11.433	4.090
700	3.193	3.127	2.971	5.453	12.443	11.290	10.179	3.632
800	2.905	2.842	2.697	4.943	11.263	10.201	9.179	3.267
900	2.668	2.608	2.471	4.525	10.295	9.309	8.361	2.969
1000	2.470	2.412	2.282	4.174	9.483	8.562	7.677	2.721

To facilitate further calculations, we characterize the energy dependence of the rate coefficients using a double-exponential function:


(4) \begin{eqnarray*}
R(E) = {a_1} \cdot {e^{{b_1} \cdot E}} + {a_2} \cdot {e^{{b_2} \cdot E}} + c,
\end{eqnarray*}

where *a*_1_, *a*_2_, *b*_1_, *b*_2_ and *c* are fitting parameters, and *E* represents the electron energy. This fitting approach effectively captures the behavior of radiative recombination rate coefficients (Fig. S2).

To determine optimal truncation of the principal quantum number *n*, we performed a comprehensive convergence analysis for Xe^52+^, varying *n* from 6 to 13 (Fig. S3). Although higher *n* states contribute to the total recombination rate, their influence diminishes beyond certain thresholds [15,16]. At medium electron energy of 500 eV, the rate coefficients show a clear convergence pattern, with variations of 0.61%, 0.92% and 1.20% between *n* = 13 and *n* = 10. Below *n* = 10, the changes become pronounced, reaching 1.45%, 2.13% and 4.75% from *n* = 9 to *n* = 6. Further inclusion of higher *n* states provides diminishing returns in accuracy, and fitting parameters convergence analysis demonstrates stable behavior for *n* ≥ 10. Furthermore, truncating at *n* = 10 strikes an optimal balance between computational efficiency and accuracy, with an error of < 2% for energy ≥ 500 eV. Consequently, *n* = 10 was adopted as the truncation threshold for calculating all cross sections and rate coefficients of Xe ions.

### Photon energy distribution at an electron energy of 500 eV

**Figure 2.**
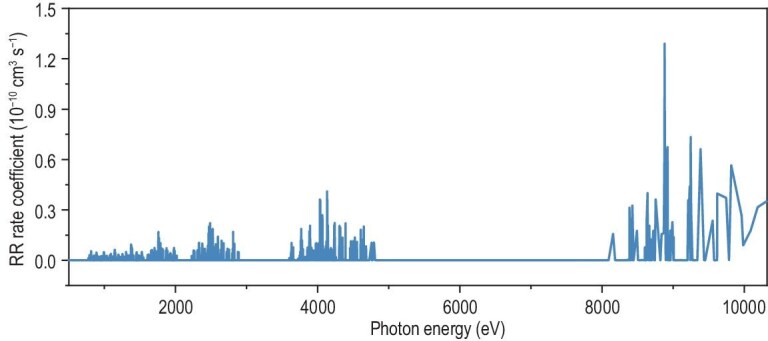
Radiative satellite spectra with electron energy of 500 eV. The radiative recombination rate coefficient represents the probability of emitted photons with different energies.

Using the obtained rate coefficients, the corresponding photon energy distribution at an electron energy of 500 eV is calculated (Fig. 2). The rate coefficients represent the photon emission probability at specific energies, with higher rate coefficients indicating more probable radiative recombination channels. This distribution reveals three distinct emission regions: a dominant hard X-ray region in 8–10 keV and two weak soft X-ray regions in 1–3 keV and 3.5–5 keV. The prominent hard X-ray emission arises from free electrons being captured into low principal quantum number orbitals of highly charged Xe ions. These emission features are advantageous for energy conversion due to the narrow spectral distribution of photon energies. Several conversion approaches have the potential to align with these well-defined photon energy ranges (1–10 keV) for acceptable energy conversion efficiency. Thermoelectric conversion using radiation-resistant materials and multilayer photoelectric devices designed for specific energy ranges are promising. A hybrid strategy integrating both mechanisms may provide a route toward enhanced conversion efficiency.

### Time evolution of the radiative recombination process

The temporal evolution of the radiative recombination process in electron-rich environments under the 500 eV electron condition is investigated to characterize the fundamental behavior of highly charged Xe ions. The density of ions at a given charge state *n_i_(t)* changes over time due to two competing processes: (i) recombination from a higher charge state *i + 1* to *i*; (ii) recombination from the charge state *i* to a lower charge state *i − 1*.

**Figure 3.**
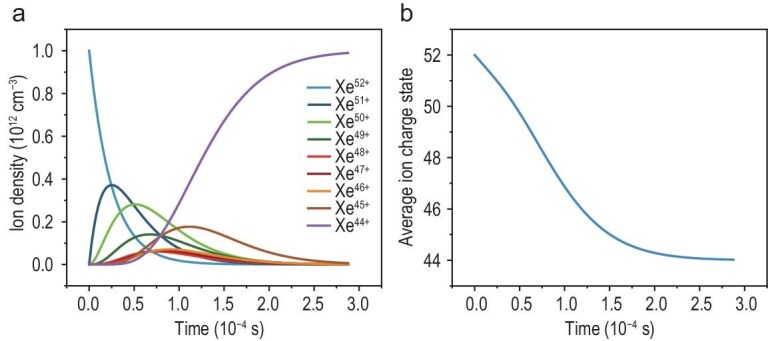
(a) Time-dependent densities of different charge states from Xe^52+^ to Xe^44+^. (b) Temporal evolution of average charge state over time. The system reaches the equilibrium state by 2.88 × 10⁻^4^ s.

The initial Xe^52+^ ion density is set at 10^12^ cm^−3^, while a typical electron density of 10^14^ cm^−3^ is selected to represent the electron beam output in experimental facilities. As shown in the time-dependent population densities of various charge states from Xe^52+^ to Xe^44+^ (Fig. 3a), the Xe^52+^ population rapidly decreases, leading to the sequential formation of lower charge states. Each intermediate charge state from Xe^51+^ to Xe^45+^ exhibits similar characteristic behavior, with an initial increase followed by a decrease. The peaks of intermediate charge states represent the sequential dominance of radiative recombination processes at different time intervals. For instance, Xe^51+^ reaches its maximum density of 0.37 × 10^10^ cm⁻^3^ at around 0.28 × 10⁻^4^ s, followed by similar sequential peaks for lower charge states. The gradual accumulation of Xe^44+^, reaching the initial ion density by 2.88 × 10⁻^4^ s, indicates the completion of the primary energy release phase. The evolution of the average charge state over time reveals the timescale of the energy release process (Fig. 3b). A rapid change occurs during the first 10⁻^4^ s, after which the rate of change slows, with the average charge state asymptotically approaching 44+ by 2.88 × 10⁻^4^ s. The characteristic time of 2.88 × 10⁻^4^ s, representing the duration required for the radiative recombination process following a single injection of highly charged ions, demonstrates significant potential for rapid and high-power output applications. Continuous and stable energy release can be achieved through sustained injection of highly charged ions, while the power output can be precisely regulated by controlling beam parameters.

### Total power output and energy release

Based on the above investigation of the recombination process, we further analyze the power output and energy release of highly charged Xe ions. The power density depends on several factors, including electron energy distribution, individual transition cross sections, ion and electron density, and interaction volume.

**Figure 4.**
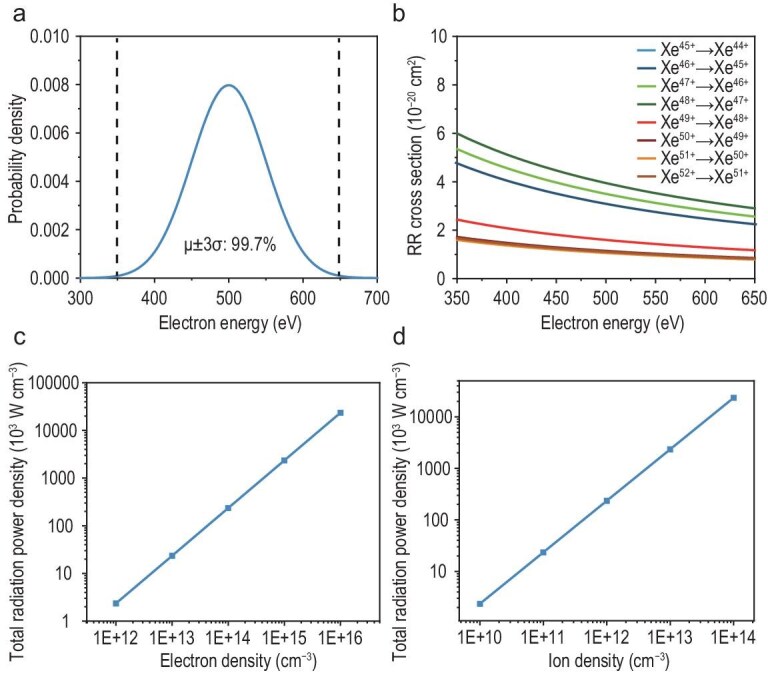
(a) Electron energy distribution function *f(E)* characterized by a Gaussian profile (*μ* = 500 eV, *σ* = 50 eV). (b) Single-exponential fits of radiative recombination cross sections ${\sigma _{RR}}( E )$ for xenon ions (Xe^52+^ to Xe^45+^) as a function of electron energy. (c) Total radiation power density under various electron densities (*n_i_* = 10^12^ cm^−3^). (d) Total radiation power density under various ion densities (*n_e_* = 10^14^ cm^−3^).

The electron energy distribution is modeled as a Gaussian profile centered at 500 eV with a standard deviation of 50 eV (Fig. 4a). The resulting radiative recombination cross sections *σ_RR_*(*E*) exhibit a consistent exponential decay with energy (Fig. 4b), which can be described by:


(5) \begin{eqnarray*}
{\sigma _{RR}}(E) = a \cdot {e^{b \cdot E}},
\end{eqnarray*}

where *a* and *b* are fitting parameters, and *E* represents the electron energy.

The total radiation power density exhibits linear dependence on both electron and ion densities. As the electron density increases from 10^12^ to 10^16^ cm⁻^3^ and the ion density from 10^10^ to 10^14^ cm^−3^, the radiation power density scales from 2.35 × 10^3^ W⋅L^−1^ to 2.35 × 10^8^ W⋅L^−1^ (Fig. 4c and d). For practical considerations, we selected moderate density parameters where the electron density (n_e_ = 10^14^ cm⁻^3^) is readily achievable using conventional electron sources, and ion density (n_i_ = 10^12^ cm⁻^3^) is comparable to typical beam intensities in current electron cyclotron resonance ion sources (ECRIS) facilities [17]. Under these conditions, the system generates a power density of 2.35 × 10^5^ W⋅L^−1^. Using the calculated characteristic time of 2.88 × 10⁻^4^ s for the considered radiative recombination process, we have obtained a mass energy density of 8.65 × 10^7^ Wh⋅kg^−1^.

## CONCLUSION AND PERSPECTIVE

In conclusion, we present the feasibility of utilizing inner-shell electron transitions for energy conversion. Direct electron capture into inner shells during radiative recombination of highly charged Xe ions from Xe^52+^ to Xe^44+^ can release photons in the 8–10 keV range, with a remarkable radiation power density of 2.35 × 10^5^ W⋅L^−1^ and an exceptional energy density of 8.65 × 10^7^ Wh⋅kg^−1^. In short, we herein report a promising solution to exceed the power and energy density limitations of current electrochemical storage technologies.

These theoretical studies could be further verified by many experimental technologies. Highly charged ion production and confinement may be made by various devices such as ECRIS, which typically operate with a microwave power of 1–2 kW. Additional technical options include electron beam ion traps (EBITs), with their precise charge state control, and laser ion sources (LISs), capable of generating high peak currents in pulsed mode. The precise control of ion injection and magnetic confinement ensure stable operations. Looking forward, the development of these high-power- and high-energy-system-utilizing inner-shell electrons will require systematic integration and optimization. A key challenge lies in the efficient utilization of output radiation energy at the 1–10 keV range. The optimization of both energy input processes and output conversion efficiency will be crucial for achieving favorable energy profit ratio (EPR). Meanwhile, standard radiation safety protocols need to be implemented following well-established practices in existing radiation facilities.

Both opportunities and challenges lie ahead. The combined high power and energy density could revolutionize fields requiring intense power outputs in confined spaces, from emergency response to space exploration and specialized industrial processes. Through systematic optimization of energy efficiency and component integration, we aim to develop a practical energy system that harnesses the exceptional potential of inner-shell electrons.

## METHODS

Detailed calculations are available in the onlines upplementary data.

## Supplementary Material

nwaf139_Supplemental_File
