# Supplementary material for: Inner-shell electrons enable both high power and energy densities
Source: Natl Sci Rev. 2025 Apr 10;12(6):nwaf139. doi: 10.1093/nsr/nwaf139 (PMC12063093; doi:10.1093/nsr/nwaf139)
Supplement: nwaf139_Supplemental_File [file nwaf139_supplemental_file.pdf]

Supporting Information

Inner-shell electrons enable both high power and energy densities

*Yi Jiang, Kaiwen Zeng, Zhe Yang, Tianrui Li, Yunting Zhang, Bingjie Wang, and Huisheng Peng**

State Key Laboratory of Molecular Engineering of Polymers, Department of Macromolecular Science, Institute of Fiber Materials and Devices, and Laboratory of Advanced Materials, Fudan University, Shanghai 200438, China.

**Correspondence and requests for materials should be addressed to Huisheng Peng (penghs@fudan.edu.cn).*

This file includes:

Calculation Methods (Pages S2-S4)
Supplementary Figures 1 to 3 (Page S5-S7)

Calculation Methods

Time evolution of recombination process

A set of rate equations are constructed to track the temporal evolution of different ion charge states. The transition rate is proportional to the free electron density n_e, the ion density in the higher charge state n_i, and radiative recombination rate coefficient $\alpha_{i\rightarrow i-1}$:

$$R_{i\rightarrow i-i} = n_e \cdot n_{i-1} \cdot \alpha_{i\rightarrow i-1} \quad (1)$$

The rate equation for the ion density in state *i* is thus given by:

$$\frac{dn_i}{dt} = R_{i\rightarrow i-1} - R_{i-i+1} \quad (2)$$

We assume that initially, at time $t = 0$, all ions are in the highest charge state. Thus, the initial condition can be described as:

$$n_{52}(0) = n_{\text{ions}}, n_{51}(0) = n_{50}(0) = \cdots = n_{44}(0) = 0 \quad (3)$$

The total number of ions is conserved throughout the processes:

$$n_{52}(t) + n_{51}(t) + \cdots + n_{44}(t) = n_{\text{ions}} \quad (4)$$

To quantify the overall charge state of the ion population at any given time, we calculate the mean charge state *q(t)*, defined as the weighted average of the charge states, expressed as:

$$q(t) = \frac{\sum_{i=44}^{52} q_i \cdot n_i(t)}{\sum_{i=44}^{52} n_i(t)} \quad (5)$$

By solving these equations, the distribution of ions in each charge state at any time, as well as the time evolution of the mean charge state can be calculated.

Radiation power density and energy density

The following calculating approach is used to accurately predict both instantaneous power output and total energy yield from the system under various operating conditions. The total radiative recombination rate characterizes the overall frequency of recombination events across all charge states. Thus, all possible transition channels are summed. The equation is expressed as:

$$\dot{N}_{RR} = [\Sigma_Z \alpha_{RR}(Z)] \times n_e \times n_i \times V \quad (6)$$

where n_e is the electron density, n_i is the ion density, $\alpha_{RR}(Z)$ represents the radiative recombination rate coefficient for charge state Z, and V is the plasma volume.

For each charge state, the radiative recombination rate coefficient $\sigma_{RR}(Z)$ is defined as the statistical average of radiative recombination cross sections and relative velocity:

$$\alpha_{RR}(Z) = \langle \sigma_{RR}(Z) v_{rel} \rangle = \int_0^\infty \sigma_{RR}(Z,E) v_{rel}(E) f(E) dE \tag{7}$$

where $\sigma_{RR}(Z)$ is solely dependent on electron energy, and v_{rel} is the relative velocity of ion and electron.The electron energy distribution $f(E)$ can be described by various distribution functions, such as Maxwell-Boltzmann for thermal plasmas, mono-energetic distributions for precisely controlled electron beams, and Gaussioan distribution for common electron beam systems, depending on specific experimental requirements.

Therefore, considering the electron energy distribution, the complete expression for the radiative recombination rate becomes:

$$\dot{N}_{RR} = \Sigma_Z \left[\int_0^\infty \sigma_{RR}\,(Z,E) v_{rel}(E) f(E) dE \right] \times n_e \times n_i \times V \tag{8}$$

To simplify the model, we assume that ion velocities are negligible compared to electron velocities. The relative velocity can be approximated by the electron velocity, which can be calculated with the energy of electrons. The equation is express as:

$$\dot{N}_{RR} = \Sigma_Z \left[\int_0^\infty \sigma_{RR}\,(Z,E) \sqrt{\frac{2E}{m_e}} f(E) dE \right] \times n_e \times n_i \times V \tag{9}$$

The average photon energy for each charge state is calculated using cross section weighted averaging:

$$E_{photon}(Z) = \frac{\Sigma_i [\sigma_i(Z,E) \times E_{photon}(Z,i,E)]}{\Sigma_i \sigma_i(Z,E)} \tag{10}$$

Finally, the radiative power density per unit volume P_{rad} is given by:

$$P_{rad} = \dot{N}_{RR} \times E_{photon} \tag{11}$$

Based on our calculation of transition kinetics, the total radiative energy per unit volume E_{total} is calculated as:

$$E_{total} = P_{rad} \times t \tag{12}$$

where t is the duration of the radiation process. The energy density is determined by dividing the total radiative energy E_{total} by the total mass of the system, including total mass of Xe ions and electrons.

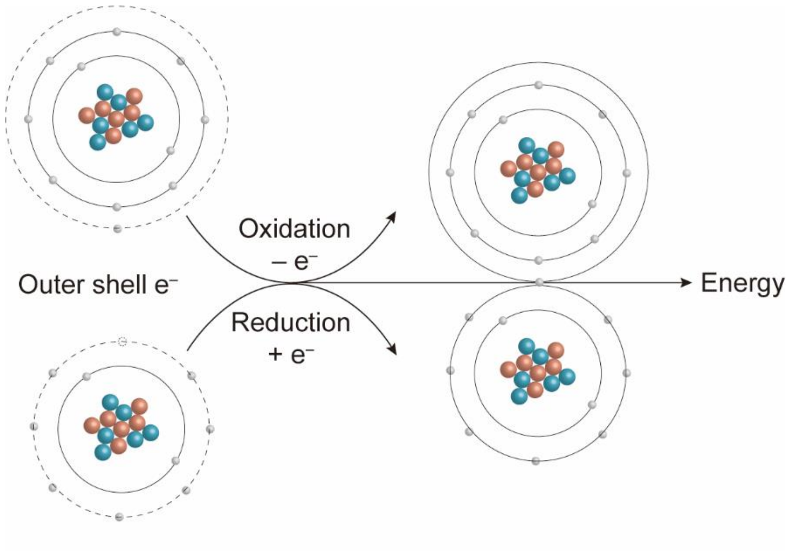

Figure S1. Schematic diagram of chemical reaction.

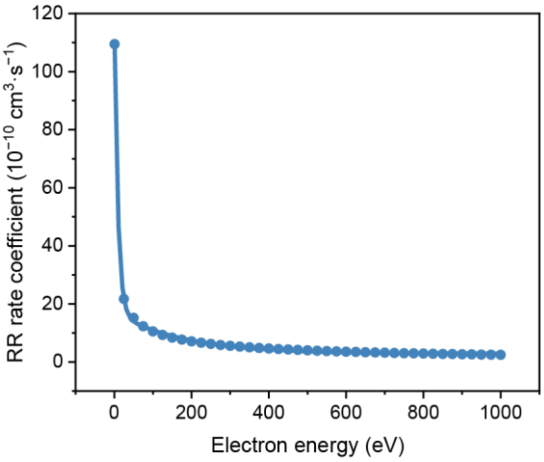

Figure S2. Calculated radiative recombination rate coefficient of Xe^{52+} and the double-exponential fitting curve. Here n = 10.

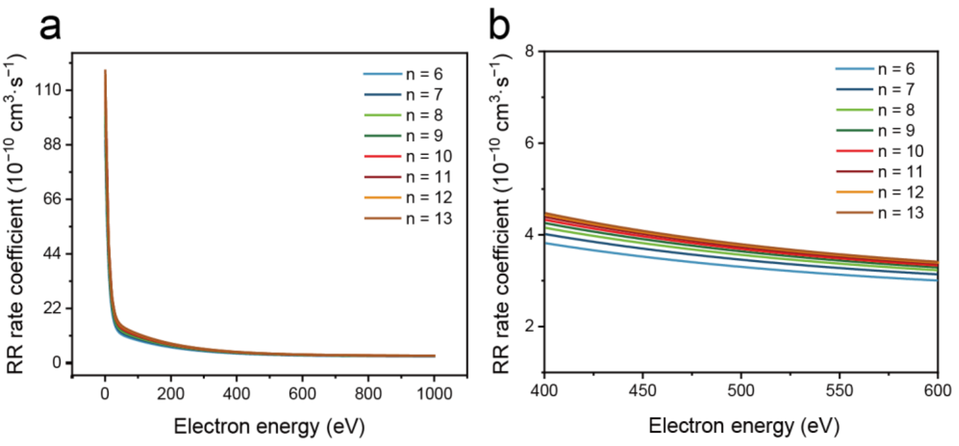

Figure S3. (a) Fitting curves of radiative recombination rate coefficient (Xe^{52+}) with principal quantum numbers of 6 to 13. (b) Magnified energy region of interest (400–600 eV).
